# Cardiovascular Diseases Increased among the Rural and Urban Population of the Northern Regions of the Republic of Kazakhstan during COVID-19: A Descriptive Study with Forecasting

**DOI:** 10.31083/j.rcm2503100

**Published:** 2024-03-08

**Authors:** Kulbayeva Shynar, Seiduanova Laura, Berdesheva Gulshara, Suleimenova Roza, Sadykova Assel, Yerdenova Maral

**Affiliations:** ^1^Department of Clinical Disciplines, Kokshetau University named after Sh. Ualikhanov, 020000 Kokshetau, Republic Kazakhstan; ^2^Department of Health Policy and Management, NJSC “Kazakh National Medical University named after S.D. Asfendiyarov”, 050000 Almaty, Republic Kazakhstan; ^3^Department of General Hygiene, NJSC “West Kazakhstan Medical University named after M. Ospanov”, 030012 Aktobe, Republic Kazakhstan; ^4^Department of Public Health and Hygiene, NJSC “Astana Medical University”, 010000 Astana, Republic Kazakhstan; ^5^Department of Clinical Disciplines, Al-Farabi Kazakh National University, 050040 Almaty, Republic Kazakhstan; ^6^ Department of Epidemiology with a course on HIV infection, NJSC “Kazakh National Medical University named after S.D. Asfendiyarov”, 050000 Almaty, Republic Kazakhstan

**Keywords:** cardiovascular diseases, urban health, rural health, ambulatory care, forecasting

## Abstract

**Background:**

The biggest health problem in most developed countries of the world, including Kazakhstan, is high morbidity and death rates due to cardiovascular diseases (CVD), both in urban and rural areas. As is known during the outbreak of COVID-19, the inaccessibility of many medical services played a big role in the incidence of CVD, in particular in the northern regions of the Republic of Kazakhstan (KZ). The objective of our research was to analyze the prevalence of CVD in city and village regions of the northern regions of the Republic of Kazakhstan, considering the outbreak period with forecasting.

**Methods:**

A descriptive study with forecasting was conducted based on the “Health of the population of the Republic of Kazakhstan and the activities of healthcare organizations”, secondary statistical reporting data (collected volume) of the KZ. Information from this database was collected for five districts, two cities and one city of regional significance in the northern region of the KZ.

**Results:**

According to our descriptive study, the incidence of CVD indicates a comparatively large prevalence of CVD among the municipal population of the northern regions of the KZ. The prevalence of CVD in urban areas of the North Kazakhstan region (NKR) was 1682.02 (2015) and 4784.08 (2020) per 100,000 population. Among rural NKR residents, it was (per 100,000 population) 170.84 (2015) and 341.98 (2020). According to the forecast, by 2025, the incidence of CVD will grow, both in urban (7382.91/100,000) and in rural areas (417.29/100,000).

**Conclusions:**

Given the situation during the pandemic, the incidence of CVD has had a sharp increase, both in the rural and in urban areas of the northern regions of the KZ. This may be due to the poor availability of medical facilities, and medical services, which may have prevented timely diagnosis, as well as the psychology of the situation and the load on cardiac activity in relation to the pandemic.

## 1. Introduction

According to the World Health Organization (WHO), more than 10 million people die from cardiovascular disease (CVD) every year, which is about 30% of all worldwide deaths [1]. Kazakhstan (KZ) ranks first in mortality from CVD out of all the states in the European Union, Central and Eastern Europe, as well as the Central Asian region [2]. According to the indicators from the WHO, the death level of the population of the KZ from cardiovascular diseases is 2 times higher than in other states. This indicator was driven by an epidemiological transition consisting of industrialization, urbanization, and associated lifestyle changes that primarily affected developed countries and spread secondary to developing countries [3, 4]. Due to the rapidly growing burden of non-communicable diseases, especially CVDs, health policy has meanwhile been reoriented to the real epidemiological situation during COVID-19. Emerging epidemic trends in CVD in our region have not received due attention.

Favorable mortality rate from CVD in our country developed with the implementation of the Programme for the Development of Cardiological and Cardio surgical care in KZ for 2007–2009. Additionally, this was developed using the State Healthcare Programme of the KZ, “Salamatty Kazakhstan/Healthy Kazakhstan” for 2011–2015, the State Health Development Programme of the Republic of Kazakhstan “Densaulyk/Health” for 2016–2019, the State Health Development Programme of the Republic of Kazakhstan for 2020–2025. These state programme consider new reform of the healthcare system in the KZ, including the improvement of the basic work of cardiology, interventional and cardiac surgery medical services; the use of successful techniques of rehabilitation, early detection, care and therapeutic prevention of patients and people with disabilities with CVD; professional development and retraining of professionals in the field of cardiology, etc. [5, 6, 7]. However, the northern or outlying regions remain without due attention from the state.

The level of education differed between communities, and the proportion of people with higher formal education was lower in rural areas than in urban areas. Many studies have documented a close relationship among socio-economic parameters (education, profession, income, marital status) and the development of cardiovascular diseases in countries with various socio-economic statuses, as well as among rural or adult populations. Only a few studies have addressed this issue in countries with economies in transition, such as the countries of the past Soviet Union, such as the Republic of Kazakhstan [8].

At the same time, the psycho-emotional state of the patient greatly affects the cardiovascular system due to COVID-19 [9]. Indeed, pre-existing CVD has been associated with a worse prognosis and more severe COVID-19 progression and complications, while COVID-19 itself can also cause arrhythmia, cardiac involvement, fulminant myocarditis, heart failure, pulmonary embolism, and disseminated intravascular disease and coagulation. Due to the various unknown and long-term consequences, an ongoing need for cardiovascular risk assessment should also be considered in all patients who survive COVID-19 [10].

In our country, the distribution of medical workers across the territory is not even: this is due to the higher density of medical workers in urban areas due to better living conditions and, of course, higher wages compared to rural areas. About 40% of the country’s population lives in rural areas, and such patients have a higher prevalence of persistent physical illnesses requiring medical intervention, but at the same time, access to specialized services may be difficult for this category of the population. Given that access to specialized medical care is currently a top priority in healthcare management, understanding the geographical distribution and provinciality of this population in relation to medical centers can help in the coordination of medical care. In addition, the poor quality of roads, and the lack of developed infrastructure lead to the lack of equal and fair access of the population to all levels of medical care [11, 12, 13].

Given the above, epidemiological patterns and changes in CVD need to be characterized in order to prioritize public health tools. In this regard, the purpose of this study was to demonstrate up-to-date information on the epidemiological situation for CVD, assess their chronological trends over a certain period, as well as assess their prospective prognostic trends in the northern regions, taking into account the population in comparison with other regions of the Republic of Kazakhstan.

## 2. Methods

This was a descriptive study based on indicators obtained from the state statistical reporting “Health care and the activities of healthcare organizations in the Republic of Kazakhstan” [14]. These analyzes of data were first calculated using descriptive statistics for study variables. These statistics bring together a group of data, providing in-depth information about the sample and showing information about the population from which the sample was drawn. From this database, we obtained data on the incidence of CVD from 2015–2020. At the same time, a calculation was made to predict the incidence of CVD until 2025. For the forecasting calculation, data including population numbers and the incidence of CVD, taken from the database was used.

Predictive modeling uses statistics and known results to process and create models that can predict future outcomes with some degree of accuracy.

Regression analysis is a basic approach to forecasting time series of any nature, easily implemented using any computer mathematics system. Non-adaptive models make it possible to obtain morbidity projections for any period [15].

Non-adaptive regression models are designed to take into account the entire history of morbidity in the analyzed territory. To construct them, all available data or, at least, observations of recent years that have similar characteristics are used.

The study also used a descriptive research method, which includes creating a frequency table, and calculating statistical characteristics or graphical presentation.

### 2.1 Collecting Process

Statistical reporting “Healthcare and the activities of health organizations of the Republic of Kazakhstan” is presented annually and represents the collected volume. This statistical collection encompasses statistical materials on the activity of healthcare organizations and health indexes of the population of the Republic of Kazakhstan for different years, and also contains data on CVD, grouped by doctors employed in main healthcare institutions throughout the country. The data collection procedure is standardized and obligatory for any primary-care physician.

These regions were chosen because they considered the difficulty of obtaining first aid during the pandemic. When using the data, we selected the morbidity rate of the adult population for comparison between city and village patients with CVD.

**Fig. 1.**
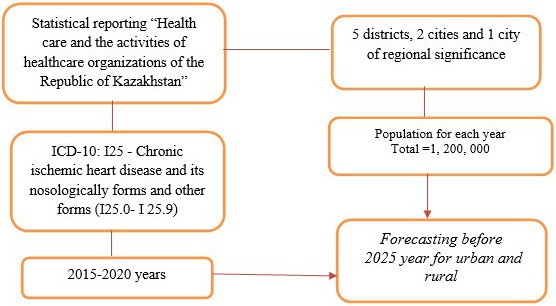
**Flow chart for sample size selection**. ICD, International Classification of Diseases.

The northern region divided into 5 districts, 2 cities and one city of regional significance, the population in these regions is approximately 1,200,000 people (Fig. 1).

### 2.2 Inclusion Criteria

We selected all patients with chronic CVD who are registered at the clinic. Data were taken from the database of these health institutions. This study included the adult population registered with the dispensary with a diagnosis according to International Classification of Diseases (ICD)-10: I25 - Chronic ischemic heart disease and its nosologically forms and other forms (I25.0–I25.9). In addition, the study included patients with chronic cardiovascular diseases living in the northern regions and registered in clinics for cardiovascular disease (3 cities, 5 districts) of the KZ. According to the methodology of long-term modeling and forecasting of the development of diseases, building a predictive trend line in these categories (urban and rural).

### 2.3 Statistical Analysis

General incidence rates calculated per 100,000 people.

The total incidence rate calculated as the ratio of the total number of diseases associated with CVD to the estimated population:

The total number of recent cases of cardiovascular diseases during one indicated year

In all ages/average annual total population × 100,000 people.

All calculations performed using the statistical package for social science software, SPSS.20 (IBM Corp., Chicago, IL, USA).

There is another value on the graphs, obtained because of trending ($R^{2}$). The coefficient of determinism designates how successful the resulting regression model is (0 to ±1).

The dynamics of indicators of cardiovascular disease indicators was studied over 5 years, with this incidence trend, you can use least squares methods, the formula:

y = a + bx

where: y—equalized indicator;

x—a conventional series of numbers, symmetrically located relative to zero;

a—conditional average;

b—competition coefficient.

The study protocol was reviewed and approved by the Local Ethical Commission of NJSC “Astana Medical University” (No. 4 dated 30.03.2022).

## 3. Results

According to the study results, cardiovascular diseases increased among the rural and urban population of the northern regions of the KZ during the COVID-19 period.

Table 1 shows the incidence of the population of the northern region of the KZ, registered in the healthcare institutions from 2015 to 2020. In 2015, the incidence among the urban population was 3574, in 2020-10747 (+3 times growth dynamics). In rural localities, the incidence of CVD was 766, in 2020-1568 (the dynamics of growth by 2 times).

**Table 1.** Morbidity of the population in the northern regions of the KZ, registered with the health organization*.

Years	Population (abs. number)		Morbidity (abs. number)		Per 100,000 population	
	Urban	Rural	Urban	Rural	Urban	Rural
2015	212,483	44,836	3574	766	1682.02	170.84
2016	215,560	45,533	4586	815	2127.48	178.99
2017	216,442	45,639	5218	841	2410.81	184.27
2018	218,493	45,363	6415	905	2936.02	199.50
2019	219,094	44,980	8095	925	3694.76	205.65
2020	224,641	45,851	10,747	1568	4784.08	341.98

*Northern regions are included 5 districts, 3 cities. KZ, Kazakhstan.

Also, for the indicated years, the northern region of KZ with information on how much more people fell illness, as can be seen from Table 1, in urban areas, the morbidity of CVD remains high compared to rural areas. Although the population is much smaller. In urban areas (100,000 population), the incidence of CVD was 1682.02 in 2015 (in 2016 - 2127.48; in 2017 - 2410.81; in 2018 - 2936.02, in 2019 - 3694.76) and in 2020 year was 4784.08. This means that the incidence rate of CVD in urban areas has tended to increase every year, especially in 2020 during quarantine measures, the incidence of CVD had a sharp increase in incidence.

In rural areas of Kazakhstan (100,000 population), the incidence of CVD was 170.84 in 2015 and during the subsequent years, the growth dynamics were not sharp (2016 - 178.99; 2017 - 184.27; 2018 - 199.50; 2019 - 205.65); in 2020 it was 341.98. In this category, the population is smaller than in the city, but the incidence of CVD is also high.

**Fig. 2.**
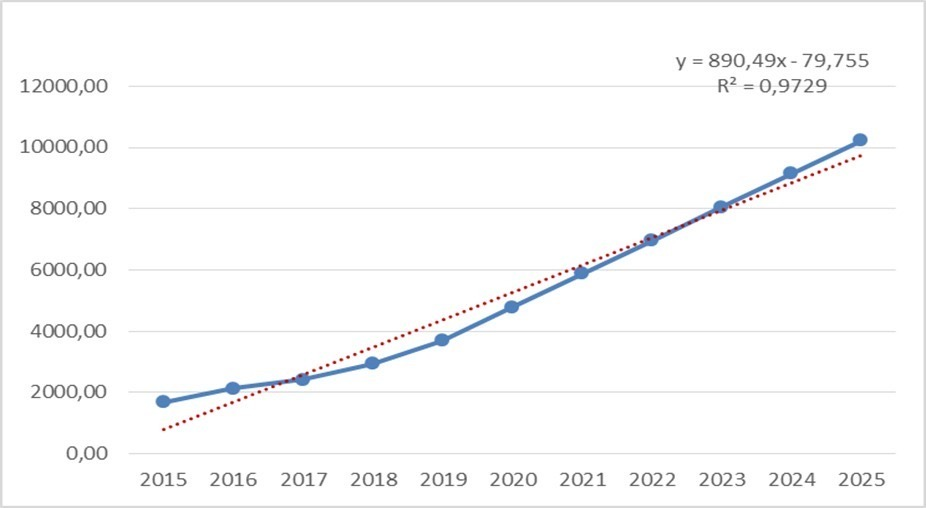
**The incidence of cardiovascular disease (CVD) in urban areas, taking account forecasting**.

The predictive measures of the expected incidence of CVD in the entire urban population of the northern region of the Republic of Kazakhstan in 2023 will be 6197.92/100,000, by 2024 6790.42/100,000 and by 2025 they will be 7382.91/100,000 (Fig. 2).

There is a trend of constant growth from 2017 to 2025, the regression linear scale goes up ($R^{2}$ = 0.9729, y = 890.49x – 79.755). According to the calculations of long-term modelling and forecasting of the development of diseases, by constructing a predictive trend line in this category, the regression model is correct.

**Fig. 3.**
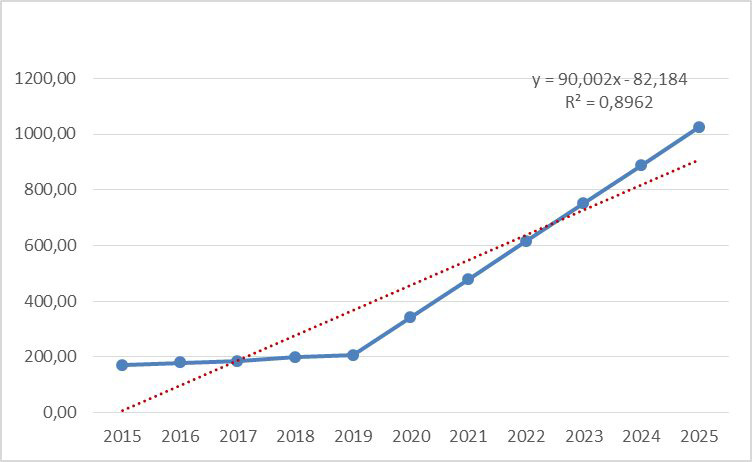
**The incidence of CVD in rural areas, considering forecasting**.

In rural areas of the northern region of the Republic of Kazakhstan in 2023 it will be equal to 335.79/100,000, by 2024 - 390.13/100,000 and by 2025 - 417.29/100,000 (Fig. 3).

Moreover, here it should be noted that the growth trend from 2019 to 2025, the regression linear scale is directed upwards ($R^{2}$ = 0.8962, y = 90.002x – 82.184), respectively. According to the calculations of long-term modelling and forecasting of the development of diseases, by constructing a predictive trend line in this category, the regression model is correct, as in the urban one.

According to the results of a study in urban areas, the incidence of CVD among the adult population from 2015 to 2020 tends to constantly increase, and according to our calculations, there will be a sharp increase from 2020 to 2025. In rural areas, this indicator was stable until 2019 and there was no increase in incidence, but from 2020 there was a sharp increase until 2025. The cause-and-effect relationship is that despite the high and emergency assistance to the population in the city, the economic characteristics of the city, environmental factors and, of course, the number of populations compared to the village have a large influence. In villages in the northern regions, clean air, organic nutrition, and a healthy lifestyle showed the stability of CVD disease.

Based on the results obtained, it can be concluded that in urban conditions the quality of medical care provided is much better than in rural areas. This may be due to the lack of highly specialized personnel, not timely provision of emergency assistance, etc.

During the pandemic, timely receipt of medical services at the outpatient level was not available; the high increase in incidence is associated with the results of quarantine measures and long-term isolation of the population. In general, the country is not ready to use remote or online medical services in this period.

## 4. Discussions

It was believed that rural residents make up 40% of the population of the Republic of Kazakhstan [16]. The majority of the rural population lives in poor living conditions (especially poor-quality roads), communications and the lack of modern means of communication, the Internet, in general, indicates a very low level of their access to medical organizations. According to statistics, the appealability of rural residents to medical institutions is 2.5 times lower than that of urban residents [17]. It should be noted the high prevalence among the rural population of chronic non-communicable diseases (chronic diseases of the circulatory system, respiratory organs, blood, diabetes mellitus), which occupy a leading place in the structure of disability and mortality in all countries of the world [18].

In many countries, there is a health disparity between rural and urban areas, and cardiovascular disease is no exception. Although numerous international studies report worse outcomes for the rural population, this is not the case in Kazakhstan, where the rural population tends to have lower rates of morbidity and mortality [19]. Perhaps this is due to the low availability of medical facilities, which is typical for the rural areas of our country, and the lack of timely access to medical care, and diagnostics, which can interfere with the successful treatment process.

It should be noted that today Kazakhstan is aiming to further reducing mortality from CVD and has developed a new regulatory document “Human health and the healthcare system” and proposed a new national public health programme for 2020–2025. This programme was based on evidence-based demographic strategies and cost-effective data that can cover very large populations, and includes legislative measures for the prevention of noncommunicable diseases. Improved health information systems, such as surveillance of morbidity, mortality and disability, are necessary to justify health policy and set targets, as well as to continuously monitor the results of intervention programme [20].

At the same time, it is necessary to consider new quarantine measures around the world and in our country, to develop new methods and approaches to providing medical care at the outpatient level. In addition, the significant psychological impact of the pandemic on patients. Recent evidence suggests that the rate of hospitalization for acute heart disease during the COVID-19 pandemic is much lower than expected, indicating limited access to emergency medical services due to fears of infection in hospitals and therefore the importance of mobile health care in this context, both in the city and in the countryside. Epidemiological studies documenting home and hospital deaths during the COVID-19 epidemic compared to previous years over the same period will likely clarify this [21, 22].

Considering the increase in morbidity rates both in the city and in the countryside, there is an urgent issue of developing and implementing mobile applications for remote monitoring, which can be effective both for patients themselves and for medical workers, which will improve not only the quality of service, but also ensure its effectiveness and efficiency. Smartphones are actively used today.

Since the pandemic, research in the field of remote medical services has increased significantly in our country. The need for these online services in emergency situations such as a pandemic [23, 24]

Remote monitoring of patients is one of the key international areas in the field of healthcare informatization. This is due to an increase in the proportion of elderly people, an increase in the number of chronic diseases, an overload of outpatient clinics, and patient dissatisfaction assistance provided to them.

Thus, there is a need for prospective cohort studies to establish and explain this phenomenon. In addition, both urban and rural populations of Kazakhstan can benefit from the adaptation of preventive strategies to reduce cardiovascular diseases, given our forecast.

## 5. Conclusions

In many countries, there are differences in health status between rural and urban areas, and cardiovascular disease is no exception. Given the situation during the pandemic, the incidence of CVD has a sharp increase, both in the rural and in the urban in the northern regions of the KZ.

Assessing changes in the dynamics of CVD incidence is important for healthcare providers to plan and manage cardiac care. At the same time, they, as well as epidemiologists, are faced with the question of to what extent, for example, the increase in morbidity is due to the “ageing” of the population and to what extent is it due to an increase in the risk of getting sick due to the emergence of new or intensification of existing socio-economic and epidemiological factors. Of course, the problem can be formulated in this definition if during the period under study in this population there have been no significant changes in the state of registration and the quality of diagnosis.

This may be due to the poor availability of medical facilities, and medical services, which prevented timely diagnosis, as well as the psychological situation and the load on cardiac activity. Economic and social factors also play a role. Given the results of CVD forecasting, it is necessary to develop and implement new reforms in this area.

## Data Availability

All data points generated or analyzed during this study are included in this article and there are no further underlying data necessary to reproduce the results.
